# Mitochondrial Peptide Humanin Protects Silver Nanoparticles-Induced Neurotoxicity in Human Neuroblastoma Cancer Cells (SH-SY5Y)

**DOI:** 10.3390/ijms20184439

**Published:** 2019-09-09

**Authors:** Sangiliyandi Gurunathan, Muniyandi Jeyaraj, Min-Hee Kang, Jin-Hoi Kim

**Affiliations:** Department of Stem Cell and Regenerative Biotechnology, Konkuk University, Seoul 05029, Korea; muniyandij@yahoo.com (M.J.); pocachippo@gmail.com (M.-H.K.)

**Keywords:** silver nanoparticles, humanin, cell death, oxidative stress, mitochondrial dysfunctions, DNA damage, apoptosis

## Abstract

The extensive usage of silver nanoparticles (AgNPs) as medical products such as antimicrobial and anticancer agents has raised concerns about their harmful effects on human beings. AgNPs can potentially induce oxidative stress and apoptosis in cells. However, humanin (HN) is a small secreted peptide that has cytoprotective and neuroprotective cellular effects. The aim of this study was to assess the harmful effects of AgNPs on human neuroblastoma SH-SY5Y cells and also to investigate the protective effect of HN from AgNPs-induced cell death, mitochondrial dysfunctions, DNA damage, and apoptosis. AgNPs were prepared with an average size of 18 nm diameter to study their interaction with SH-SY5Y cells. AgNPs caused a dose-dependent decrease of cell viability and proliferation, induced loss of plasma-membrane integrity, oxidative stress, loss of mitochondrial membrane potential (MMP), and loss of ATP content, amongst other effects. Pretreatment or co-treatment of HN with AgNPs protected cells from several of these AgNPs induced adverse effects. Thus, this study demonstrated for the first time that HN protected neuroblastoma cells against AgNPs-induced neurotoxicity. The mechanisms of the HN-mediated protective effect on neuroblastoma cells may provide further insights for the development of novel therapeutic agents against neurodegenerative diseases.

## 1. Introduction

Humanin (HN) is a novel 24-amino acid peptide that is highly conserved across species, and it is secreted from cells and found in circulation, as well as bound to cell membranes; its actions are mediated by specific receptors [1,2]. HN is found in many cell types such as the endothelial cells [3] and germ cells [4], and is also detectable in plasma [5]. HN plays critical role in various biological processes including apoptosis, cell survival, lipid flux, and inflammation [6,7]. Several studies demonstrated the cytoprotective effects of HN in various type of cells such as the neurons [8,9], pancreatic β cells [10], testicular germ cells, Leydig cells [11], and GH3 cells [12]. In addition to its cytoprotective effects, HN exerts antiapoptotic activity by modulating the intrinsic mitochondrial pathway and expression and intracellular location of the Bcl-2 family members like Bax and Bcl-2 [13]. HN interacts with proapoptotic Bcl-2 family members and interferes with their translocation to mitochondrial proteins such as Bax and Bid, thereby inhibiting formation of the apoptosome and activation of caspase-3 [2,14]. For instance, the mitochondria-dependent intrinsic pathway is a signaling pathway indispensable for germ cell apoptosis across species post hormonal deprivation [15]. HN protects neurons against oxidative stress-induced damage from oxygen-glucose deprivation, hypoxia-induced cell death, and cerebral infarction in vitro and in vivo [16]. Gotoh et al. [17] demonstrated that HN acts as a protective factor against a variety of insults including oxidative stress, serum starvation and hypoxia. For example, HN protects retinal pigment epithelial cells (RPE) cells against oxidative stress-induced cell death by enhancing mitochondrial biogenesis and bioenergetics [14] and recently Sreekumar et al. [18] reported that HN potentially protects RPE cells against oxidative stress–induced cell death and also restore mitochondrial function.

Among various metallic nanoparticles, silver nanoparticles (AgNPs) are one of the most commonly used nanoparticles (NPs) in consumer products including food storage, medical devices, and textiles. They also have numerous environmental applications [19,20], and they have also been exploited as an antibacterial, antifungal, antiviral, anticancer or antiangiogenic and so on due their unique physical and chemical properties [20]. Frequent and long-term exposure of humans to AgNPs can cause adverse effects leading to organ failure [21]. For instance, AgNPs exhibit a longer half-life in the brain particles and their related dissolved ions and this property of theirs is able to potentially cause neurotoxicity in rat brains [22,23,24]. Studies demonstrated that AgNPs induce acute intracellular calcium and consequently induce oxidative stress response in mixed primary neuronal cells [21]. AgNPs induces toxicity and neuronal cell death in primary rat cortical cell cultures by modulating cytoskeleton components, by perturbing pre- and postsynaptic proteins, and by mitochondrial dysfunction [25]. AgNPs have multiple effects, for example, AgNPs induce neuronal differentiation in human neuroblastoma cells [26,27] and AgNPs induce cell death through generation of reactive oxygen species, which is a major cause of nanoparticles-induced cell death in a variety of cancer cells including human breast cancer cells [28], human lung cancer cells [29] and human ovarian cancer cells [30]. Besides these, AgNPs induce cytotoxicity, alterations in signaling pathways [31,32], mitochondrial dysfunction [33], production of reactive oxygen species (ROS), depletion of various antioxidants, increased lipid peroxidation and accumulation of autophagosomes [34], inflammasome formation [35], apoptosis, and necrosis [36]. Frequent exposure to AgNPs from different products leads to an accumulation of AgNPs in the body and subsequently in the brain after crossing the blood brain barrier (BBB) [37,38].

The endoplasmic reticulum (ER) is a critical organelle for the synthesis and folding of membrane-bound proteins. The disruption of changes in calcium (Ca 2^+^) homeostasis, redox status, and energy stores causes dysfunction of ER and leads to ER stress [39]. ER stress promotes apoptosis through the activation of ER-specific cysteine protease, caspase-12 [40]. AgNPs induce ER stress and activate the unfolded protein response (UPR)-dependent apoptotic pathway [41,42]. ER stress and UPR activate important proteins such as the inositol requiring protein 1 (IRE1), PKR-like endoplasmic reticulum kinase (PERK), and activating transcription factor-6 (ATF-6) which act as stress sensing proteins and are early markers for ER stress induced nanotoxicity [43]. Prolonged exposure of C57Bl/6 male mice to AgNPs causes contextual cognition and behavior anomalies in mammals [44]. Neurotoxicity induced by nanoparticles causes adverse effects on the structure, function or chemistry of the nervous system by increasing level of ROS, elevation of cytokines, and dysregulation of apoptosis leading to neuronal death [45,46]. AgNPs induce neurotoxicity through the increased generation of ROS, by activation of caspases, cytokine secretion, by inducing damage to the cell membrane integrity and by causing cell necrosis [47]. Although several studies have addressed the neurotoxic effect of AgNPs, there is a considerable lack of information regarding the protective effect of HN on AgNPs-induced neurotoxicity in neuronal cells. Hence, this study aimed to investigate the protective effect of HN on AgNPs-induced cell viability, proliferation, membrane integrity, oxidative and nitro-oxidative stress, impairment of antioxidant system, mitochondrial dysfunctions, DNA damage and apoptosis in the human neuroblastoma cell line SH-SY5Y.

## 2. Results and Discussion

### 2.1. Synthesis and Characterization of AgNPs Using Delphinidin

**Figure 1:**
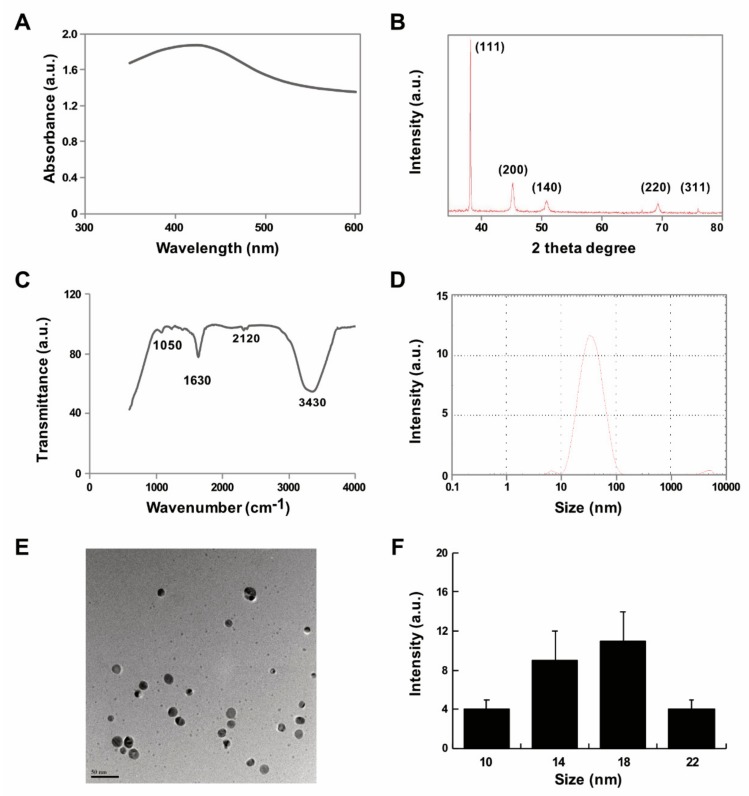
Synthesis and characterization of AgNPs using delphinidin (**A**) Absorption spectra of delphinidin-mediated synthesis of AgNPs (**B**) X-ray diffraction patterns of AgNPs (**C**) FTIR spectra of AgNPs (**D**) size distribution analysis of AgNPs by DLS (**E**) TEM micrograph images of AgNPs (**F**) Corresponding particle size distribution histograms (mean ± standard deviation) (Scale bar—50 nm). At least three independent experiments were performed for each sample and reproducible results were obtained. The data present the results of representative experiments.

Synthesis of AgNPs is carried out using different type of methods such as physical, chemical, and biological methods. Physical methods generate enormous amounts of heat and radiation. Chemical methods produce lots of toxic materials hazardous to living beings. Therefore, an environmentally friendly, easy, facile, and green method was adopted. However, when biological systems such as bacteria, fungi and plant extracts are used for synthesis of nanoparticles, the downstream processing and purification of nanoparticles from the mixture takes a longer time because of high amounts of impurities in these biological systems. Therefore, use of purified biomolecules that are pure, clean and free of impurities are better alternatives as they require less time for downstream processing. Therefore, we exploited the use of a purified biomolecule, delphinidin, for synthesis of AgNPs, which is an anthocyanin compound. Anthocyanins are a group of natural compounds and secondary metabolites belonging to the flavonoid family [48]. Synthesis of AgNPs was carried out by mixing 1.0 mg/mL delphinidin with 1 mM AgNO_3_ followed by incubation at 40 °C for 6 h. The bio-reduction of silver ion and delphinidin was confirmed by color change from yellow to brown and further spectrometric analysis showed the intensity of the surface plasmon resonance band (SPR) exhibited a maximum at 420 nm; this characteristic peak indicates the reduction of silver nitrate into AgNPs using bio reductant (Figure 1A). Similarly, an extract of saffron which contains rich anthocyanins assisted in the synthesis of AgNPs [49].

Next, the cubic nature, size, phase identification and crystalline nature and purity of AgNPs were confirmed by XRD. The diffracted intensities were recorded from 20 to 80 degrees. As shown in Figure 1B, the XRD image showed five intensely strong Bragg reflections at 39.55, 47.35, 54.75, 69.74, and 79.85 which correspond to 111, 200, 142, 220, and 311 planes, respectively, for silver (JCPDS card number 04-0783). The XRD pattern of synthesized AgNPs was in agreement with an earlier report on synthesis of AgNPs using Panchakavya, which contains five products from cows: milk, curd, ghee, urine, and dung [50]. The calculated average crystallite of the AgNPs using Debye-Scherrer formula is ~18 nm [51]. The sharp and high intensity peak of 111 indicates that the synthesized AgNPs were pure and clean. The bio-reduction of silver ions using delphinidin was confirmed by FTIR, which is an important and valuable tool for the identification of functional groups and interactions between molecules [52]. The bands observed at 3430 and 2120 cm^−1^ were assigned to the C-H stretching vibrations of the primary and secondary amines, respectively. The sharp absorption peaks at 1630 and 1050 cm^−1^ (Figure 1C) could be due to N–H stretching in primary and secondary amines and amides. The peaks in the range of 3200–3500 cm^−1^ were assigned as OH stretching in alcohols and phenolic compounds with strong hydrogen bonds [53].

To estimate the size and size distribution of synthesized AgNPs in water, we performed dynamic light scattering (DLS) analysis. The size of the particles exhibited a range between 10 and 100 nm; however the average size of particles was 30 nm, which is larger than the size we predicted from XRD and TEM (Figure 1D). The larger size of particles when detected by DLS was due to Brownian motion. Furthermore, in order to confirm the particle size in dry samples, TEM images were captured under high vacuum conditions. TEM micrographs of the AgNPs revealed distinct, uniformly and significantly distributed spherical shapes. The average particle size was estimated from various TEM images and showed particle sizes between 10 and 22 nm with an average size of 18 nm (Figure 1E,F). Similarly, biological templates such as *Datura stramonium* leaf extract with an average size of 18 nm were used. The size of the particles played an important role in cellular responses and cell toxicity. Particularly smaller sized particles exhibited greater response against cells and penetrated easily into the cells too.

### 2.2. Dose-Dependent Effect of HN and AgNPs on SH-SY5Y Cells

**Figure 2:**
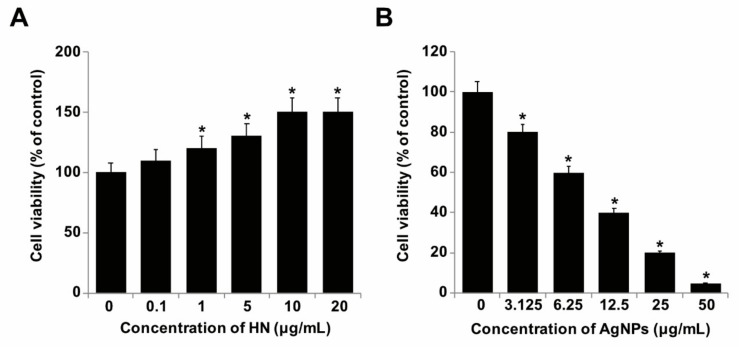
Dose-dependent effect of HN and AgNPs on cell viability of SH-SY5Y cells. (**A**) The viability of SH-SY5Y cells was determined after 24-h exposure to different concentrations of HN (0.1–20 µg/mL) (**B**) The viability of SH-SY5Y cells was determined after 24-h exposure to different concentrations of AgNPs (3.125–50 µg/mL). At least three independent experiments were performed for each sample. The results are expressed as the mean ± standard deviation of three independent experiments. Differences between the treated and control groups were measured using Student’s *t*-test. Statistically significant differences between the treated and control group are indicated by (* *p* < 0.05).

Since HN exhibited potential antiapoptotic and neuroprotective effects, we were interested in studying the cytoprotective and antiapoptotic effects of HN on AgNPs-induced toxicity in SH-SY5Y cells. To determine the protective effect of HN against AgNPs, SH-SY5Y cells were treated with various concentrations of HN and AgNPs. HN induced a dose-dependent positive effect on SH-SY5Y cells (Figure 2A). Increasing doses of HN promoted cell viability and a significant effect was observed from 1–10 μg/mL. Cells treated with 1, 5, 10 and 20 μg/mL of HN increased by 120 ± 3.08, 130 ± 4.08 and 150 ± 0.08) percent, respectively (*p* < 0.05). Similarly, increasing concentrations of AgNPs from 3.125, 6.25, 12.5, 25.0 to 50 μg/mL decreased the cell viability of SH-SY5Y cells by 20% (1.11 ± 0.20), 40% ((1.13 ± 0.35), 60 ((1.29 ± 0.80), 80 (1.2 ± 0.10 and 95% (1.71 ± 0.70), respectively (*p* < 0.05). The cell viability was significantly affected even at lower doses (Figure 2B). The half maximal inhibitory concentration (IC50) of AgNPs against SH-SY5Y was 10 μg/mL. It is well known that AgNPs have a potential reduction effect on cell viability in a variety of cancer and non-cancer cells including human breast cancer cells [28], human lung cancer and non-cancer cells [54], human ovarian cancer cells [32], human neuroblastoma cancer cells [33] and human cervical cancer cells [53]. Besides these effects, AgNPs potentially induce dose-dependent cytotoxicity in various types of neuronal cells such as embryonic neural stem cells (NSCs) from human and rat fetuses [55]. A study reported that 20 nm AgNPs not only inhibited the sprouting of neuronal branches and elongation of neurites but also caused degeneration of neuritic processes or aberrant aggregations of cell bodies by a reduction in synaptic proteins, cytoskeletal integrity, mitochondrial functionality and cell viability in a dose-dependent manner [25]. AgNPs can impair cell functions and promote cell death in hippocampal neurons [56]. Kim et al. [57] reported that AgNPs can induce significant cytotoxicity in cultured cerebral cortical neurons in a dose-dependent and time-dependent manner. Together, all these findings suggest long-term usage of AgNPs could cause serious effects on cell viability.

### 2.3. Dose-Dependent Effect of HN Restored Cell Viability that Was Decreased by AgNPs Treatment

**Figure 3:**
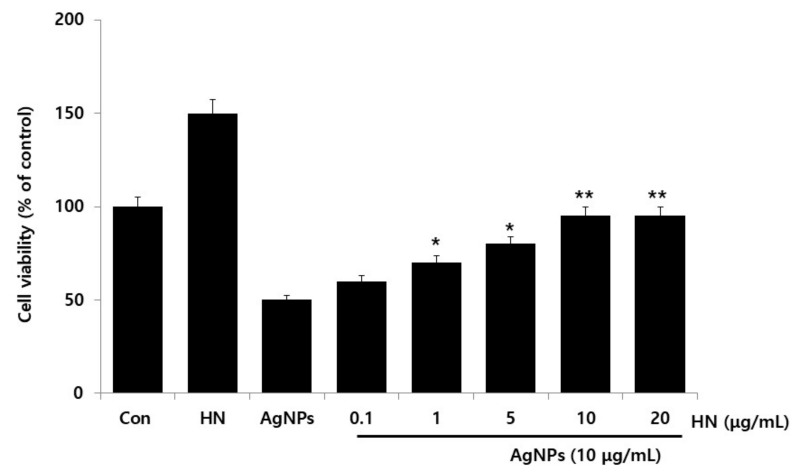
Dose dependent effect of HN on the AgNPs-induced cellular toxicity triggered increase in the cell viability of SH-SY5Y cells. The viability of SH-SY5Y cells was determined after 24-h exposure to different concentrations of HN (0.1–20 µg/mL) at a fixed concentration of AgNPs (10 µg/mL). At least three independent experiments were performed for each sample. The results are expressed as the mean ± standard deviation of three independent experiments. Differences between the treated and control groups were measured using Student’s *t*-test. Statistically significant differences between the treated and control group are indicated by (* *p* < 0.05, ** *p* < 0.01).

AgNPs were found to be toxic to SH-SY5Y cells at an IC50 of 10 µg/mL and caused a 50% decrease of cell viability in comparison to the untreated SH-SY5Y control cells as measured by CCK-8 viability assay. HN itself in the medium did not inhibit the cell viability and instead it promoted the cell viability. To determine the optimal concentration of HN that was required to overcome the toxic effects of AgNPs, cell viability assays were performed. The cells were rescued from cell death by the addition of HN in AgNPs (10 μg/mL) treated cells. When the concentration of HN reached 1.0 μg/mL, the decrease of cell viability began to attenuate in AgNPs-treated cells. Ninety-five percent of SH-SY5Y cells recovered from AgNPs-induced cell-death in cells pretreated with 10 μg/mL of HN (Figure 3). However, further increasing concentrations of HN did not show any increase in its ability to recover SH-SY5Y cells from AgNPs-induced cell death at concentrations higher than 10 µg/mL. The increasing concentration of HN from 0.1 to 10 μg/mL in 10 μg/mL-treated cells linearly rescued the cell death from 10 to 50% (1.63 ± 0.29) (*p* < 0.05). Our results indicated that 10 μg/mL of HN is optimal and a physiologically viable concentration to overcome AgNPs-(10 μg/mL of HN) induced toxicity in SH-SY5Y cells. Humanin G (HNG) protects Aβ25–35-induced loss of cell viability and dysfunctions of mitochondria and increases level of Cyt C in PC12 cells [58]. Cui et al. [59] reported that ninety-eight percentage of the cell viability of cortical neurons from neonatal Wistar rats was recovered when 10 μmol/L of HN was added. HN protects RPE cells from apoptosis induced by endoplasmic reticulum stress-inducers such as tunicamycin (TM), brefeldin A, and thapsigargin [60]. HN inhibits TNF- α -induced apoptosis in normal and tumor anterior pituitary cells via activation of various signaling pathways including NF- κ B activation, STAT3, JNK, Akt and MAPKs as well as Bcl-2 family members [61]. HN protects fibroblasts, cardiomyoblasts, neuronal cells, and primary cardiomyocytes from stress-induced cell death in a dose-dependent manner through the activation of chaperone-mediated autophagy (CMA) by increasing the level of heat-shock protein 90 (HSP90) [62]. The data from present findings and previous findings collectively suggest that HN could potentially ameliorate AgNPs-induced toxicity in SH-SY5Y cells. Based on these results, in the subsequent experiments, we used HN at 10 μg/mL concentration to protect SH-SY5Y from an AgNPs-(10 μg/mL) induced toxic effect; the selected concentration of HN did not induce changes in cell survival or integrity of control cells and it showed a positive effect on cell survival at equimolar concentration of AgNPs.

### 2.4. HN Prevents AgNPs-Induced Loss of Cell Viability and Proliferation of SH-SY5Y Cells

**Figure 4:**
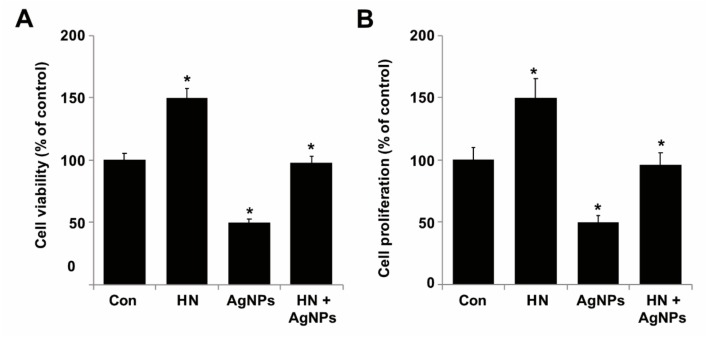
HN prevents AgNPs-induced reduction of cell viability and proliferation of SH-SY5Y cells. (**A**) SH-SY5Y cells were pretreated with or without 10 μg/mL HN for 24 h. Cells were then treated with 10 μg/mL HN and/or 10 μg/mL AgNPs for 24 h and then viability was measured using CCK-8 viability assay (**B**) SH-SY5Y cells were pretreated with or without 10 μg/mL HN for 24 h. Cells were then treated with 10 μg/mL HN and/or 10 μg/mL AgNPs for 24 h and then proliferation was measured using BrdU ELISA. At least three independent experiments were performed for each sample. The results are expressed as the mean ± standard deviation of three independent experiments. The treated groups showed statistically significant differences from the control group by the Student’s *t*-test (* *p* < 0.05).

To determine the preventive effect of HN on AgNPs-induced loss of cell viability and proliferation, SH-SY5Y cells were treated with either HN or AgNPs (10 μg/mL) separately or with a combination of both HN and AgNPs (10 μg/mL + 10 μg/mL) for 24 h. As shown in Figure 4A, pretreatment with HN (10 μg/mL) reversed AgNPs-induced toxicity by increasing survival of SH-SY5Y cells. Similarly, pretreatment with HN (10 μg/mL) reversed the AgNPs-induced decrease in proliferation efficiency of SH-SY5Y cells in comparison to the untreated group (Figure 4B). When compared to untreated cells, HN (10 μg/mL) alone promoted cell viability and proliferation up to 50% and it rescued AgNPs-induced cell death up to 48% (1.23 ± 0.40) (*p* < 0.05). Similarly, Cui et al. [59] previously reported that *N*-methyl-d-aspartate (NMDA) exposure (100 μmol/L) induced the damage of neuron cells and caused a decrease of cell viability to levels of ~53.2% (1.10 ± 0.09) in comparison to the controls, but when the cells were pre-treated with HN, the cell viability was significantly increased in rat cortical neurons by the addition of HN. Pretreatment of HUVECs with either 1 or 10 mM HN for 3 h also significantly increased the cell viability of HUVECs treated with 50 mM high glucose for 72 h. Zhao et al. [63] reported that the HN exhibited protective effects on okadaic acid-(OA) induced neurotoxicity in cultured cortical neurons. Collectively, all these results depicted that HN potentially inhibits AgNPs-induced loss of viability and cell proliferation in SH-SY5Y cells.

### 2.5. Measurement of Cell Death

**Figure 5:**
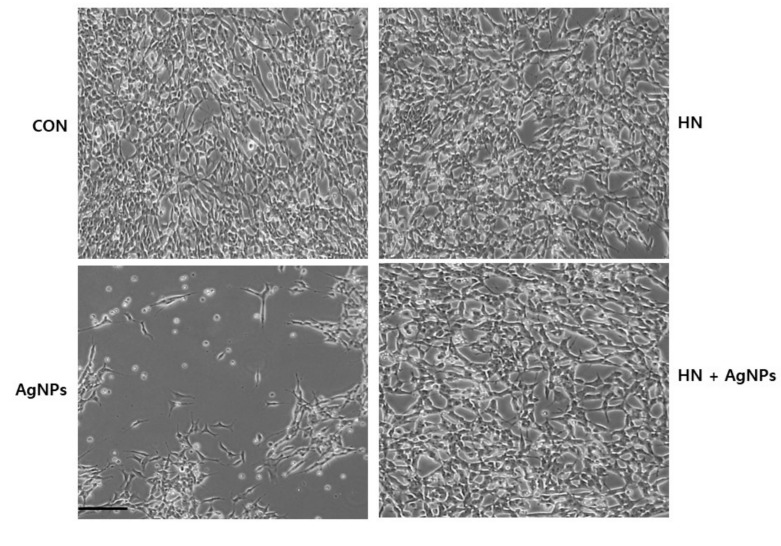
HN protects AgNPs-induced cell death. SH-SY5Y cells were pretreated with or without 10 μg/mL HN for 24 h. Cells were then treated with 10 μg/mL HN and/or 10 μg/mL AgNPs for 24 h. The cell morphology was determined using an optical microscope. Scale bar—200 µm

Morphological alterations of cells are hallmarks of apoptosis that concern both the nucleus and the cytoplasm. These morphological features can be initiated by the cleavage of various proteins by caspases and initiation of cell death; cells lose contact with neighboring cells [64]. In order to determine the protective effect of HN on AgNPs-induced altered cell morphology, SH-SY5Y cells were treated with either HN or AgNPs (10 μg/mL) separately or with a combination of both HN and AgNPs (10 μg/mL + 10 μg/mL) for 24 h; morphological changes were observed under a phase-contrast light microscope. The SH-SY5Y control group adhered to the bottom of the plate with a clear nucleus and interconnected with other cells. HN-(10 μg/mL) treated cells did not show any significant morphological differences in comparison to the control group. In contrast to HN-treated cells, AgNPs (10 μg/mL) treatment induced the cells to become round and their classical morphological characteristic features disappeared (Figure 5). Consequentially, cells detached from one another and then the detached cells burst into pieces. The changes in cell morphology by AgNPs indicated the successful induction of AgNPs-induced neurotoxicity at 100 (10 μg/mL). Interestingly, HN-pretreated cells showed HN completely reversed the deleterious effect caused by AgNPs. Similarly, NMDA caused significant alterations and damages in cell morphology of cortical neurons [59]. HN treatment inhibited (150 μM) tert-Butyl hydroperoxide-(tBH) induced senescence in human RPE cells [18]. Thus, these findings concluded that HN potentially prevents AgNPs-induced cell death.

### 2.6. HN Rescues Cells from AgNPs-induced Lactate Dehydrogenase Leakage and Dead Cell Protease Activity

**Figure 6:**
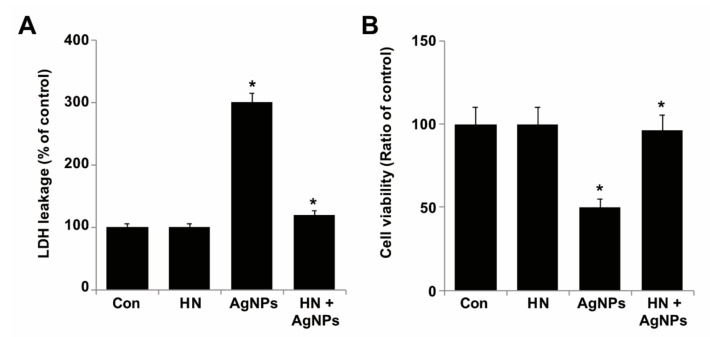
Effect of HN on AgNPs-induced leakage and dead-cell protease. (**A**) SH-SY5Y cells were pretreated with or without 10 μg/mL HN for 24 h. Cells were then treated with 10 μg/mL HN and/or 10 μg/mL AgNPs for 24 h, and the LDH activity was measured at 490 nm using the LDH cytotoxicity kit. (**B**) The level of dead-cell protease was determined by CytoTox-Glo cytotoxicity assay. The cell viability ratio was also determined according to the manufacturers’ instructions. At least three independent experiments were performed for each sample. The results are expressed as the mean ± standard deviation of three independent experiments. The treated groups showed statistically significant differences from the control group based on Student’s *t*-test (* *p < 0.05*).

Lactate dehydrogenase (LDH) is a soluble cytoplasmic enzyme which is released into the extracellular space when the plasma membrane is damaged or insulted by cytotoxic agents [65,66]. The leakage of LDH is considered to be a significant cell death marker. Although other enzymes such as adenylate kinase and glucose-6-phosphate also seem to be cell death markers, these enzymes are not stable and lose their activity during cell death assays. To determine the rescue effect of HN on the release of LDH triggered by AgNPs, SH-SY5Y cells were treated with either HN or AgNPs (10 μg/mL) separately or with a combination of both HN and AgNPs (10 μg/mL + 10 μg/mL) for 24 h and then we measured the leakage of LDH. Our results showed that AgNPs treatment (10 µg/mL) caused the damage of neuroblastoma cells and thus triggered the increased extracellular release of LDH (Figure 6). LDH concentration in the AgNPs treatment group was about 3-fold higher (300%) (1.53 ± 0.49) (*p* < 0.05) than that in the control group. As shown in Figure 6A, the addition of HN significantly reduced the release of LDH in AgNPs-treated cells. Our results demonstrated that HN itself had no direct effect on LDH in SH-SY5Y cells. However, the release of LDH was significantly inhibited by HN in AgNPs-treated cells when the cells were pretreated with HN for 24 h, HN rescued almost 46% (0.63 ± 0.19) (*p* < 0.05). Similarly, when the neurons were pretreated with HN for 16 h, HN inhibited the release of LDH in NMDA-treated cells [59]. Primary cortical neuron cells released LDH dose-dependently when treated with various concentrations of okadaic acid. When challenged with various concentrations of HN from 5 to 20 µM, the results showed a differential effect on LDH leakage; a low concentration of (5 µM HN) did not protect neurons from OA-induced toxicity (10 nM). [63]. Another study by Yang et al. [67] reported that HN diminished NMDA-induced neuronal insults by increasing cell viability, decreasing lactate dehydrogenase (LDH) release, and increasing survival of primary cortical cells that were isolated from Wistar rats. In order to confirm the cell membrane integrity, we performed an estimation of the dead cell protease markers, which can be measured using the CytoTox-Glo cytotoxicity assay kit, which can distinctively quantify the extracellular activity of an intracellular protease (dead-cell protease). These markers are associated with cell death or viability. Cell viability was calculated according to methods described earlier [68]. To calculate cell viability, luminescence was measured before and after lysis. As we expected, the ratio of cell viability was significantly reduced in AgNPs-treated cells, whereas HN prevented AgNPs-induced loss of cell viability in HN-pretreated cells (Figure 6B). Together, these results suggest that HN ameliorates AgNPs-induced leakage of LDH and the extracellular activity of an intracellular protease. 

### 2.7. HN Protects Against AgNPs-induced Rate of Reactive Oxygen Species (ROS) Production, Malondialdehyde (MDA), Nitric Oxide (NO) and Carbonylated Protein in SH-SY5Y Cells

**Figure 7:**
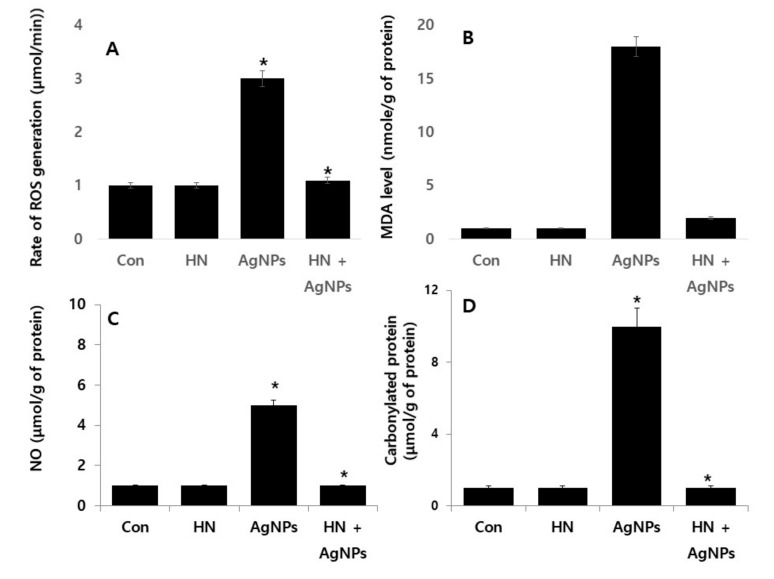
HN inhibits AgNPs-induced ROS generation, lipid peroxidation, nitric oxide and protein carbonyl content in SH-SY5Y cells. SH-SY5Y cells were pretreated with or without 10 μg/mL HN for 24 h. Cells were then treated with 10 μg/mL HN and/or 10 μg/mL AgNPs for 24 h. (**A**). ROS generation was measured in micromoles per min. (**B**). The concentration of MDA was measured using a thiobarbituric acid-reactive substances assay and expressed as nanomoles per milliliter. (**C**) NO production was quantified spectrophotometrically using the Griess reagent and expressed as micromoles (**D**). Protein carbonyl content was measured and expressed as nanomoles. The results are expressed as the mean ± standard deviation of three independent experiments. The treated groups showed statistically significant differences from the control group based on Student’s *t*-test (* *p* < 0.05).

AgNPs potentially induce anti-proliferative, necroptosis, autophagy, necrosis, and apoptosis in cancer cells through oxidative stress [20,69]. ROS are essential secondary messengers in multiple signaling pathways including cell survival and cell death [70]. Oxidative stress-induced programed cell death could be associated with loss of mitochondrial membrane potential and mitochondrial dysfunction [33,54]. Excessive generation of ROS and (reactive nitrogen species) RNS cause various damages to the cells such as DNA breakage and impairment of antioxidant potential [34]. Therefore, it is essential to determine the protective effect of HN on AgNPs-induced nitro-oxidative stress, oxidative stress, lipoperoxidation and carbonylated protein in SH-SY5Y cells. SH-SY5Y cells were treated with either HN or AgNPs (10 μg/mL) separately or with a combination of both HN and AgNPs (10 μg/mL + 10 μg/mL) for 24 h and then multiple parameters were measured. AgNPs induced at least 3- (09.31 ± 0.21), 2- (0.13 ± 0.29), 5 (0.26 ± 0.39), and 10-fold (0.90 ± 0.40) increases of ROS, MDA, NO, and carbonylated protein, respectively, compared to untreated cells (* *p < 0.05*). Interestingly, SH-SY5Y cells pretreated with HN rescued the generation of ROS, MDA, NO, and carbonylated protein (Figure 7). The rate of ROS production was inhibited by HN. The rate of ROS production by AgNPs is shown in Figure 7A. Different ROS concentrations were obtained from the different treatments and expressed in micromoles per gram of protein. The ROS production level in AgNPs-treated cells (3.13 ± 0.30) was significantly greater than in the control (1.0 ± 0.10). Interestingly, HN alone did not produce any ROS, which is almost equal to untreated cells (1.16 ± 0.02). Significant (*p* < 0.05) differences were observed between the control and AgNPs-treated group.

The rate of MDA production was performed by as described in the materials and methods. The rate of MDA production by AgNPs is shown in Figure 7B. The effect of inhibition of HN on MDA production was analysed and various concentrations of MDA were obtained from the different treatment and expressed in micromoles per gram of protein. MDA production level in AgNPs-treated cells (18.0 ± 0.50) was significantly greater than in the control (1.0 ± 0.50). Interestingly, HN alone did not produce any MDA, which is almost equal to untreated cells (2.10 ± 0.08). Significant (*p* < 0.005) differences were observed between the control and AgNPs-treated group.

ROS-induced oxidative damage, such as lipid peroxidation and protein carbonylation, is associated with various neurodegenerative disorders including retinopathies [71,72,73]. The data from the present findings are consistent with previous reports which mention that tetramethylpyrazine and HN protects cells against hydrogen peroxide-induced mitochondrial ROS generation, lipid peroxidation loss of mitochondrial membrane potential, and microtubule-associated protein-2 (MAP-2) in rat retinal cells [74]. Exogenous addition of HN protects against oxidized LDL-induced oxidative stress and apoptosis in endothelial cells [3]. Sreekumar et al. (2016) [18] reported that HN rescued cells from tert-Butyl hydroperoxide-(tBH) induced oxidative stress, and mitochondrial dysfunctions in RPE cells. HN inhibited NMDA-induced oxidative stress and nitric oxide (NO) production in cultured rat cortical neurons [75]. Further, HN protected cells from ischemia and reperfusion induced a significant increase in lactate dehydrogenase (LDH) release, MDA formation in cortical neurons and HN also induced ROS generation and MDA levels in human sperm upon freeze-thaw [76]. All these data collectively suggest that HN has a potential to induce protective response to oxidative and nitro-oxidative stress, lipoperoxidation and the carbonylated protein content in SH-SY5Y cells.

### 2.8. Effect of HN and AgNPs on Antioxidant Production by SH-SY5Y Cells

**Figure 8:**
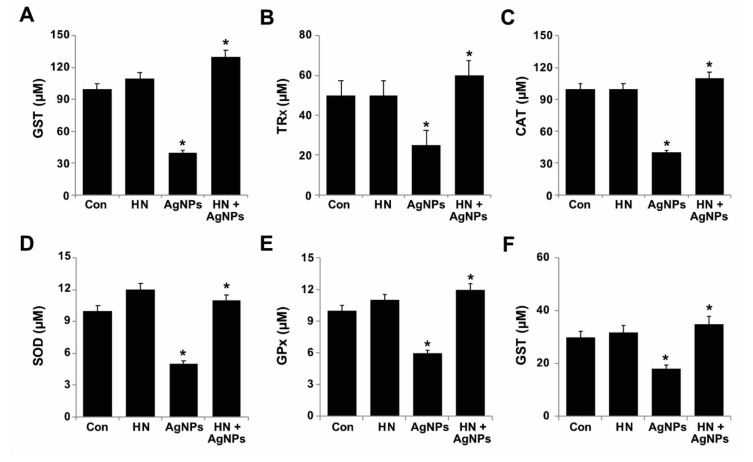
Effect of HN on AgNPs-induced reduction of antioxidant markers. SH-SY5Y cells were pretreated with or without 10 μg/mL HN for 24 h. Cells were then treated with 10 μg/mL HN and/or 10 μg/mL AgNPs for 24 h. After incubation, the cells were harvested and washed twice with an ice-cold phosphate-buffered saline solution. The cells were collected and disrupted by ultrasonication for 5 min on ice. (**A**) GSH concentration is expressed in micromoles. (**B**) TRX is expressed in micromoles. (**C**) Catalase concentration is expressed in micromoles (**D**) SOD is expressed in micromoles. (**E**) GPx concentration is expressed in micromoles. (**F**) GST concentration is expressed in micromoles. Results are expressed as mean ± standard deviation of three independent experiments. There was a significant difference in treated cells compared to untreated cells, Student’s *t*-test (* *p* < 0.05).

Increased level of ROS and decreased level of antioxidant activity are associated with the degeneration of retina and neuronal cells [77,78,79]. Redox imbalance represents an imbalance between the levels of ROS and cellular antioxidant factors. Disturbed redox homeostasis leads to harmful effects on cells [80]. ROS levels are strongly regulated by various antioxidants such as glutathione (GSH), catalase (CAT), superoxide dismutase (SOD), glutathione peroxidase (GPX), thioredoxin (TRX) (Halliwell, 2006). We were interested in investigating the protective effect of HN on AgNPs-induced reduction of antioxidant levels in SH-SY5Y cells. Thus, SH-SY5Y cells were treated with either HN or AgNPs (10 μg/mL) separately or with a combination of both HN and AgNPs (10 μg/mL + 10 μg/mL) for 24 h and then we measured the level of all the antioxidant markers. The results revealed that AgNPs significantly decreased all the tested antioxidants such as GSH (60 ± 0.2 µM), TRx (25 ± 0.7µM), CAT (60 ± 0.8 µM), SOD (4 ± 0.18 µM), GPX (4 ± 1.08 µM) and GST (12 ± 1.08 µM) compared to the control (Figure 8) (*p* < 0.05). Conversely, HN-pretreated cells rescued the effect of AgNPs on antioxidants. Zhao et al. (2012) [81] observed that pre-treatment with HN significantly increased the level of SOD activity in cortical neurons damaged by hypoxia/reperfusion (I/R). Rat cardiac myoblasts (H9C2 cells) exposed to hydrogen peroxide significantly altered the level of catalase and glutathione peroxidase (GPx). Conversely, cells treated with HNG in the presence of H2O2 induced activation of catalase and glutathione peroxidase (GPx) within 5 min and decreased the ratio of oxidized to reduced glutathione within 30 min [82]. Matsunaga et al. (2016) [60] demonstrated that pretreatment of U-251 glioma cells with HN prevented the ER stress-induced depletion of mitochondrial glutathione (GSH). In addition, co-treatment of tunicamycin (TM) and HN decreased the level of mitochondrial superoxide compared to TM treatment alone. Previously, we reported that luciferin assisted synthesis of AgNPs and graphene oxide silver nanoparticles nanocomposite reduced the levels of antioxidants proteins such as GSH, CAT, SOD, GPX and TRX in SH-SY5Y cells [27]. There was an imbalance between the oxidative and antioxidant parameters observed in brain homogenate after AgNPs treatment, with particularly increased levels of MDA concentration with a significant decrease in GSH, CAT, SOD, and GPX level of rats intoxicated with AgNPs when compared with control rats. Interestingly, the AgNPs-intoxicated rats treated with rutin significantly reversed the effect of AgNPs [83]. Dayem et al. (2018) [84] observed that AgNPs-treated cells displayed significantly decreased expression of *SOD2* and *CAT*, but no significant effects on *GPX* expression. In contrast, RA-exposed cells increase the level of antioxidant enzymes, such as *SODs, *CAT*,* and *GPX4* in SH-SY5Y cells [84]. Collectively, all these studies demonstrate that HN could protect redox imbalance induced by AgNPs.

### 2.9. HN Protects SH-SY5Y Cells from AgNPs-induced Mitochondrial Dysfunctions

**Figure 9:**
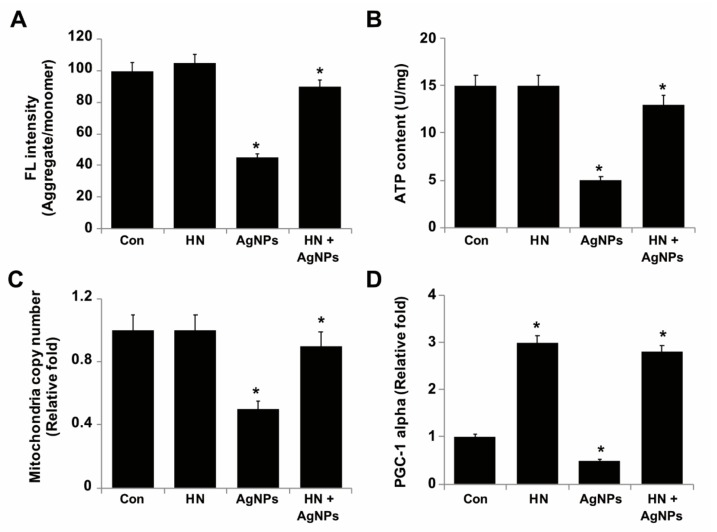
HN protects SH-SY5Y cells from AgNPs-induced mitochondrial dysfunctions. SH-SY5Y cells were pretreated with or without 10 μg/mL HN for 24 h. Cells were then treated with 10 μg/mL HN and/or 10 μg/mL AgNPs for 24 h. (**A**) Mitochondrial membrane potential (MMP) was determined using the cationic fluorescent indicator JC-1 (**B**). Intracellular ATP content was determined according to the manufacturer’s instructions (Sigma-Aldrich, St. Louis, MO, USA; Catalog Number MAK135) (**C**) Mitochondrial DNA copy number was determined by quantitative real-time PCR and was expressed relative to control cells (**D**) Expression of mRNA levels of PGC-1alpha was determined by the quantitative real-time PCR analysis. Results are expressed as mean ± standard deviation of three independent experiments. There was a significant difference in treated cells compared to untreated cells based on Student’s *t*-test (* *p* < 0.05).

Mitochondria are the primary source of energy for cells to be able to drive various cellular functions properly and thus damage to mitochondria results in decreased level of energy production. Mitochondria are the vital source of ROS generation by AgNPs and HN is involved in AgNPs-mediated induction of toxicity [29,30,54,85,86]. Mitochondrial membrane polarization is essential for ATP production and maintains the calcium level. AgNPs induce loss of Δψm, which is involved in the dysfunctions of mitochondria. Although previous studies highlight the ability of AgNPs to induce mitochondrial dysfunctions, the biological significance of HN in protection of neuronal cells from AgNPs-induced mitochondrial dysfunction remains elusive. Therefore, we aimed to evaluate the mitochondrial membrane potential, ATP content, mitochondrial DNA copy number and mitochondrial biogenesis of SH-SY5Y cells. SH-SY5Y cells were treated with either HN or AgNPs (10 μg/mL) separately or with a combination of both HN and AgNPs (10 μg/mL + 10 μg/mL) for 24 h. The results showed that AgNPs significantly induced a loss of mitochondrial membrane potential up to 55% (2 ± 0.18) in comparison to the control (*p* < 0.05), whereas HN-pretreated cells rescued AgNPs-treated cells from the loss of MMP (Figure 9A). Similarly, HN prevented H2O2 induced loss of MMP in rat retinal cell cultures [74]. Jin et al. (2010) [58] had reported that pretreatment of PC12 cells with 100 nM HNG for 6 h prior to treatment with amyloid β (25–35) significantly stabilized the mitochondrial membrane potential, blocked cytochrome C release from mitochondria and thus maintained mitochondrial functions. Pretreatment of the neurons with HN (1 μmol/L) led to a significant increase of mitochondrial succinate dehydrogenase (SDH) activity and membrane potential [75].

Next, we examined the production of ATP in the presence of either AgNPs or HN separately or pretreatment of SH-SY5Y cells with HN before treatment with AgNPs; the level of ATP production (5 ± 0.18 U/mg) was significantly reduced up to two fold in the cells treated with AgNPs, whereas pre-treatment with HN potentially rescued the effect caused by AgNPs up to the level of 13 ± 1.18 U/mg (*p* < 0.05) (Figure 9B). Excessive generation of ROS membrane depolarization of mitochondria, ATP hydrolysis and mitochondrial swelling [87]. HN analogs increased ATP levels by 92% in 30 min of exposure, with a 134% increase in 120 min in rat cardiac myoblasts [82]. Apoptotic neuron-secreted HN12 inhibited cell apoptosis in nonapoptotic cells by maintaining the function of mitochondria such as the production of ATP and the release of cytochrome C [88]. Exposure of pancreatic MIN6 β cells to HN significantly increased oxygen consumption and noticeably increased intracellular ATP levels [89].

To gain further evidence of the protective action of HN against AgNPs-induced mitochondrial dysfunctions, we estimated the relative mtDNA copy number using real-time qPCR, in which we amplified cytochrome b (Cytb) in mtDNA and actin β (ACTB) in nuclear DNA, a technique performed in a study previously in order to estimate the copy number of mitochondria [90]. The average copy number per cell was determined by the mtDNA/ACTB ratio (Figure 9C). We found that AgNPs-treated cells had lower mtDNA copy numbers 50% (2.3 ± 0.29) *p* < 0.05) compared to untreated cells which could lead to an increased level of ROS. Increased ROS level caused a major result of mitochondrial dysfunction and ROS levels are usually tightly regulated by Ras [91]. Human retinal pigment epithelial (hRPE) cells exposed to tert-Butyl hydroperoxide (tBH) causes oxidative stress induced loss of mitochondrial dysfunction by decreasing mitochondrial DNA copy number. When the cells were exposed to HN, they increased mitochondrial DNA copy number significantly up to 90% (2.5 ± 0.76) (*p* < 0.05) compared to the control group and upregulated mitochondrial transcription factor A, a key biogenesis regulator protein [18]. Finally, we aimed to address the effects of HN on AgNPs-triggered modulation of mitochondrial biogenesis in SH-SY5Y cells. Therefore, we analyzed the expression level of PGC-1 alpha, which is a central regulator of mitochondrial biogenesis in AgNPs-treated SH-SY5Y cells. The results showed that the expression level of PGC-1 alpha significantly decreased in AgNPs-treated SH-SY5Y cells (1 fold), (0.07 ± 0.05) but the levels of PGC-1 expression significantly increased in cells treated with HN alone (2 fold) (0.12 ± 0.18) or in SH-SY5Y cells pre-treated with HN and subsequently treated with AgNPs (1.8 fold) (0.16 ± 0.08) (*p* < 0.05). (Figure 9D). The data indicate that HN potentially elevates the expression of genes responsible for mitochondrial biogenesis. Similarly, Qin et al. [89] reported that HN dose-dependently increased the expression of PGC-1alpha in MIN6 β-cells. Collectively, all these findings suggest that HN potentially could rescue AgNPs-induced mitochondrial dysfunctions in SH-SY5Y cells.

### 2.10. HN Inhibits AgNPs-induced Expression of Apoptotic, Antiapoptotic and ER Stress Sensors Genes

**Figure 10:**
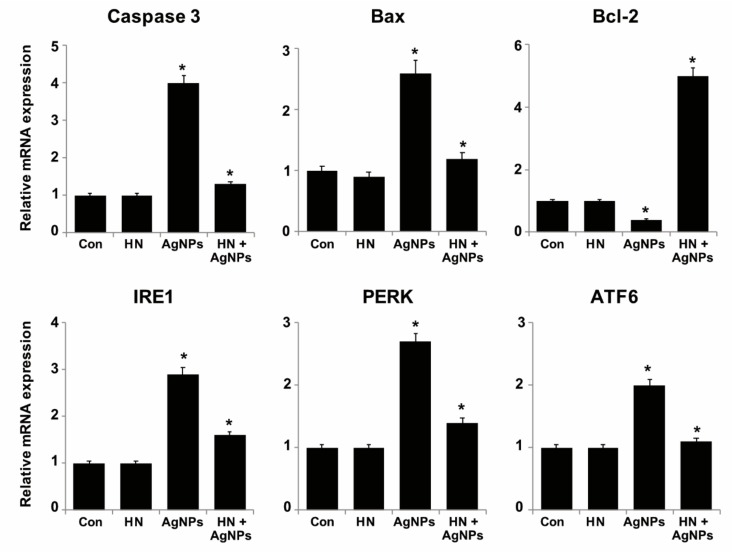
Effect of HN on AgNPs mediated modulation of the expression of apoptotic, antiapoptotic and ER stress sensor genes in SH-SY5Y cells. SH-SY5Y cells were pretreated with or without 10 μg/mL HN for 24 h. Cells were then treated with 10 μg/mL HN and/or 10 μg/mL AgNPs for 24 h. Relative messenger RNA (mRNA) expression of apoptotic, antiapoptotic and ER stress sensor genes was analyzed by quantitative reverse-transcription polymerase chain reaction. After 24 h treatment expression fold level was determined as fold changes in reference to expression values against GAPDH. Results are expressed as fold changes. Results are expressed as the mean ± standard deviation of three independent experiments. There was a significant difference in treated cells compared to untreated cells based on Student’s *t*-test (* *p*< 0.05).

Generally, AgNPs induce apoptosis by two different signaling pathways, extrinsic pathways that activate caspases or intrinsic apoptotic pathways via members of the Bcl2-protein family members [30]. The antiapoptotic protein Bcl-2 regulates mitochondrial function by inhibiting mitochondria depolarization [92]. To explore the molecular mechanisms of HN against AgNPs-induced apoptosis, SH-SY5Y cells were treated with either HN or AgNPs (10 μg/mL) or with a combination of both HN and AgNPs (10 μg/mL + 10 μg/mL) for 24 h and the expressions of key genes involved in AgNPs-induced apoptosis such as caspase 3, Bax and Bcl-2 were analyzed to determine whether the regulation of these cell death associated genes might be responsible for the protective effect of HN. As we expected, AgNPs induced expression of (3 ± 0.08), Bax (1.6 ± 0.08) and downregulated the expression of Bcl-2 (0.4 ± 0.05) significantly (*p < 0.05*). HN pretreatment prevented AgNPs-induced downregulation of Bcl-2 (5 ± 0.8) fold and upregulation of caspase 3, Bax, and thus, increased the Bcl-2/ Bax expression ratio (Figure 10). The mechanism of inhibition of Bax by HN is because of its ability to interact with the membrane-bound Bax and tBid, preventing the recruitment of cytosolic Bax and its oligomerization in the membrane. HN restored the levels of proapoptotic caspase 3, Bax and antiapoptotic Bcl-2. All these observations were in accordance with observations from previously published reports [58,93]. These HNs could prevent mitochondrial dysfunction induced by AgNPs through these effects.

ER homeostasis can be disrupted by the level of intracellular calcium (Ca 2^+^) level, redox status, and energy stores, culminating in ER stress [39]. ER stress induces the expression of three different transmembrane sensor proteins: inositol-requiring enzyme (IRE), PKR-like endoplasmic reticulum kinase (PERK) and activating transcription factor-6 (ATF-6) [94,95]. Furthermore, ER stress promotes apoptosis via activation of caspase 12 and caspase 3. Recently, studies demonstrated that AgNPs induce ER stress and activate the UPR-dependent apoptotic pathway in mammalian cells [35,41]. Despite many studies addressing the involvement of AgNPs in the induction of neurotoxicity through activation of ER stress, there is no understanding on how to overcome these ER perturbations using mitochondrial peptides. In order to confirm that the ER stress induced by AgNPs was rescued by HN, SH-SY5Y cells were treated either with HN or AgNPs (10 μg/mL) separately or with a combination of both HN and AgNPs (10 μg/mL + 10 μg/mL) for 24 h and then we measured the expression of IRE, PERK and ATF-6 genes. As shown in Figure 10, levels of IRE (1.9 ± 1.11-fold), PERK (1.7 ± 0.18-fold) and ATF-6 (1.0 ± 0.09) fold mRNA were significantly (*p < 0.05*) increased by the treatment of SH-SY5Y cells with AgNPs in comparison to the control, and pretreatment of AgNPs-treated SH-SY5Y cells with HN remarkably decreased the expression of all IRE, PERK and ATF-6. Recently, Li et al. (2018) [96] observed AgNPs can potentially induce ER stress sensors genes in SH-SY5Y cells and in turn promote apoptosis. The authors found that all the tested ER stressors induce apoptosis dose-dependently. Furthermore, pre-incubation of cells with HN attenuates ER stressor-induced apoptosis. Collectively, all these results indicate that the ER stress signaling pathways that are activated by AgNPs can be competently and potentially deactivated by HN.

### 2.11. HN Inhibits AgNPs-induced Apoptosis in SH-SY5Y Cells

**Figure 11:**
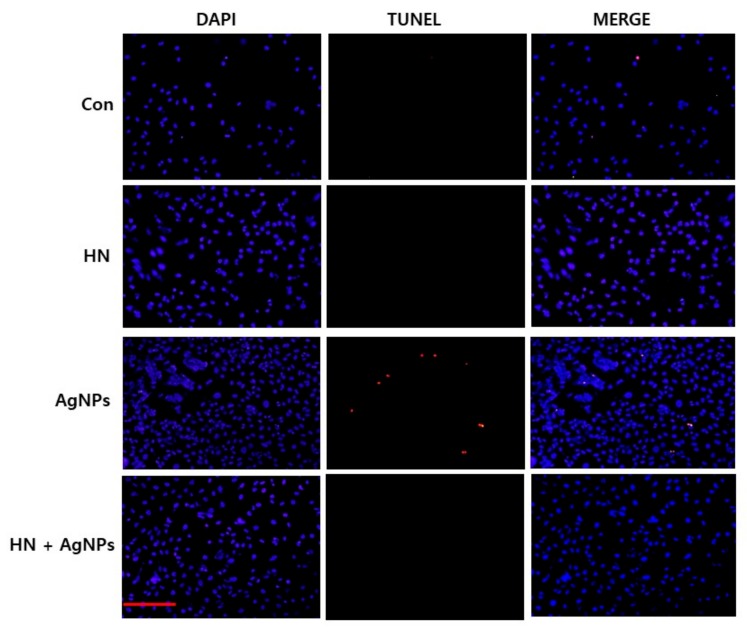
HN inhibits AgNPs-induced apoptosis. SH-SY5Y cells were pretreated with or without 10 μg/mL HN for 24 h. Cells were then treated with 10 μg/mL HN and/or 10 μg/mL AgNPs for 24 h. Apoptosis in SH-SY5Y cells after a 24-h treatment was assessed using the TUNEL assay; the nuclei were counterstained with DAPI. Representative images show apoptotic (fragmented) DNA (red staining) and the corresponding cell nuclei (blue staining). (Scale bar—100 µm)

AgNPs that interact with the cell membrane are internalized into the cells and subsequently activate signaling pathways to generate ROS. ROS production is the major mechanism of AgNPs-induced toxicity in variety of cancer cells, non-cancer cells and neuronal cells. ROS production leads to damage of proteins, lipids and nucleic acids caused by the strong affinity of silver for sulfur, finally resulting in the inhibition of cell proliferation [21,30]. AgNPs induce apoptosis by initiating various intracellular signals to activate intrinsic and extrinsic pathways and also p53 dependent and independent pathways and also by the activation of various kinases involved in death signal [30]. For example, several studies in vitro and in vivo studies confirmed that the AgNPs exhibit neurotoxic effects by inducing inflammation and increasing blood brain barrier permeability [97,98]. Although considerable studies support that AgNPs induce neurotoxicity, there is no information about how to overcome this neurotoxicity, particularly in neuroblastoma cancer cells. Therefore, we were interested in determining the effect of HN on AgNPs-induced apoptosis in SH-SY5Y cells. SH-SY5Y cells were treated with either HN or AgNPs (10 μg/mL) separately or with a combination of both HN and AgNPs (10 μg/mL + 10 μg/mL) for 24 h and apoptosis was estimated by TUNEL assay. TUNEL assay is a fast and sensitive assay to assess DNA fragmentation at the single-cell level. SH-SY5Y cells treated with AgNPs (10 μg/mL) were significantly positive for apoptotic DNA fragmentation. In control cells as well as HN treated cells, no apoptotic cells were observed. AgNPs cells pretreated with HN also showed no TUNEL-positive apoptotic cells, suggesting that HN could potentially inhibit AgNPs-induced apoptosis in SH-SY5Y cells (Figure 11). Our results are consistent with previous report by Yuan et al. (2017) [53] which demonstrated AgNPs-induced apoptosis in SH-SY5Y cells. A toxicity study revealed that AgNPs coated with ethylene oxide and citrate showed decreased cell viability, disturbed barrier integrity, and triggered perturbations in junctions, oxidative stress, and DNA strand breaks in brain [99]. Park et al. (2017) [33] observed that AgNPs inhibit brain-derived neurotrophic factor-induced cell survival through DNA fragmentation induced apoptosis in SH-SY5Y cells. Gottardo et al. (2014) [12] demonstrated the antiapoptotic effect of HN on TNF-α in anterior pituitary cells by TUNEL assay. For instance, AgNPs-induced DNA damage in various types of cells, including human ovarian cancer cells, breast cancer cells, and neuroblastoma cells [27,28,30]. Similarly, AgNPs induces oxidative DNA damage in mouse embryonic stem cells [100]. An in vivo study demonstrated the size-and dose dependent genotoxic effects of AgNPs in liver cells of Swiss albino mice [101]. The results clearly demonstrated that the pretreatment of HN (0.5 µM) completely blocked the proapoptotic effect of TNF-α in total anterior pituitary cells, lactotropes, and somatotropes from both female and male rats. In addition, HN inhibited the apoptotic effect of TNF-α on pituitary tumor cells. Matsunaga et al. (2016) [60] reported that ER stress activators induced apoptosis rescued by HN via upregulation of mitochondrial glutathione in RPE cells. Together, these data demonstrate that HN exhibits an anti-apoptotic effect against AgNPs-induced apoptosis.

### 2.12. HN Protects AgNPs-Induced Oxidative DNA Damage

**Figure 12:**
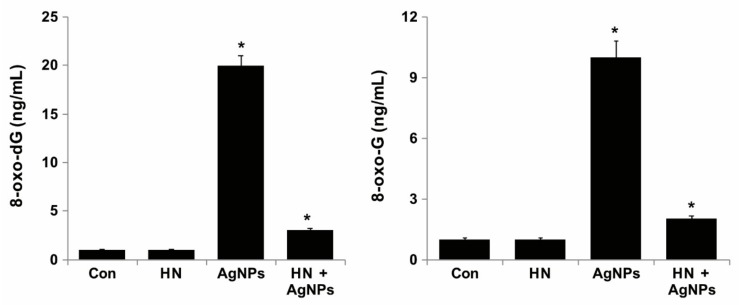
HN protects AgNPs-induced oxidative damage caused to DNA. (**A**) SH-SY5Y cells were pretreated with or without 10 μg/mL HN for 24 h. Cells were then treated with 10 μg/mL HN and/or 10 μg/mL AgNPs for 24 h. 8-oxo-dG was measured after 24 h of exposure of SH-SY5Y cells. (**B**) SH-SY5Y cells were pretreated with or without 10 μg/mL HN for 24 h. Cells were then treated with 10 μg/mL HN and/or 10 μg/mL AgNPs for 24 h. 8-oxo-G was measured after 24 h of exposure of SH-SY5Y cells. Results are expressed as mean ± standard deviation of three independent experiments. There was a significant difference in treated cells compared with untreated cells based on Student’s *t*-test (* *p* < 0.05).

One of the possible mechanisms of AgNPs-induced apoptosis is the generation of ROS which causes oxidative damage to DNA and chromosomes. Oxidative DNA damage includes oxidized DNA bases and DNA single strand and double strand breaks. Guanine is the most susceptible to oxidation by ROS resulting in the generation of 8-oxoguanine (8-oxoG). [102,103]. To address whether HN pretreatment inhibits AgNPs induced oxidative DNA damage-repair, SH-SY5Y cells were treated with HN or AgNPs (10 μg/mL) or with a combination of HN and AgNPs (10 μg/mL + 10 μg/mL) for 24 h, and the level of accumulation of 8-oxodG and 8-oxoG was estimated by ELISA. The results showed that the AgNPs-treated group showed significant accumulation of both 8-oxodG and 8-oxoG compared to the control group or HN treated group, whereas HN-pretreated cells showed significantly lower accumulation of 8-oxodG and 8-oxoG (Figure 12). AgNPs induced accumulation of 8-oxodG (20 ± 2.08 ng/mL) and 8-oxoG (10 ± 1.08 ng/mL) to several fold in SH-SY5Y cells (*p < 0.05*). This study showed that HN can lower the level oxidative DNA damage caused by AgNPs. ROS mediated accumulation of 8-oxoG is a common phenomenon in carcinogenesis and neurodegeneration. 8-oxoG levels are significantly increased in mitochondrial DNA (mtDNA) as well as nuclear DNA (nDNA) in the brains of patients with Parkinson’s disease (PD), Alzheimer’s disease (AD), and Huntington’s disease (HD) [104,105,106]. Accumulation of 8-oxoG in mitochondrial DNA resulted in excessive formation of SSBs and causes mitochondrial DNA depletion, ATP depletion, opening of the membrane permeability transition pore, and finally, mitochondrial dysfunction [107]. Accumulation of 8-oxoG in the mt DNA of neurons caused calpain-dependent neuronal loss and delayed nuclear accumulation of 8-oxoG in the microglia, resulting in PARP-dependent activation of apoptosis inducing factor and exacerbated microgliosis [108]. Recent studies have demonstrated that accumulation of 8-oxoG in mt DNA causes mitochondrial dysfunction and impairs neuritogenesis in cultured adult mouse cortical neurons under oxidative conditions [109]. HN can potentially inhibit oxidative stress-induced DNA damage and accumulation of 8-oxodG and 8-oxoG.

### 2.13. Potential Mechanism of Protective Effect of HN on Cell Viability and Cell Proliferation

**Figure 13:**
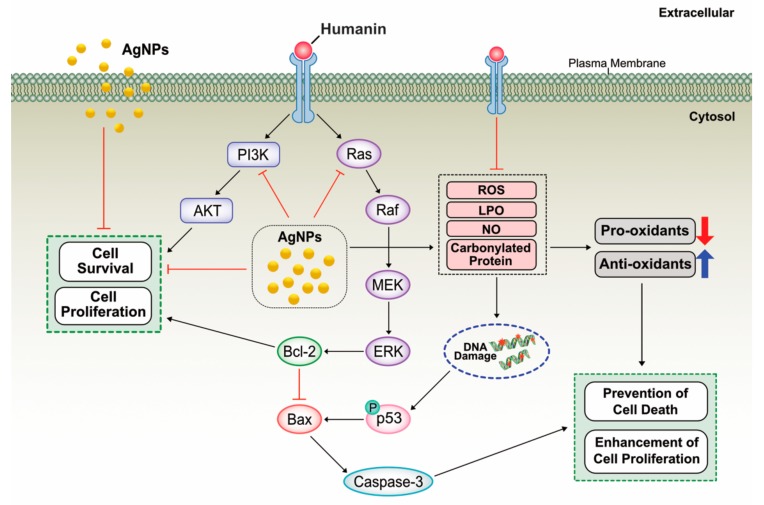
A hypothetical model describes protective effect of HN on AgNPs-induced cell death. The red “T” bar represents inhibition, the blue arrow represents up regulation, the red arrow represents down regulation, the black arrows represents activation/regulation of downstream molecules.

AgNPs are known to induce cell death through inhibition of cell viability and cell proliferation by the mechanism of inhibition of phosphorylation of cell survival kinase such as Akt, which is mainly involved in cell viability and cell proliferation. For example, AgNPs inhibit vascular endothelial growth factor (VEGF)-induced cell viability, cell proliferation, and migration by inhibition of phosphorylation of Akt in bovine retinal endothelial cells (BRECs) [110,111]. Based on our findings, the results suggest that HN could protect AgNPs-induced cell death by increasing cell viability and cell proliferation in human neuroblasoma cells. HN can function both in the extracellular and intracellular environments and it can bind and regulate a number of intracellular proteins, such as components of JAK2/ STAT3 pathway or an insulin-like growth factor binding protein 3 (IGFBP3) [112,113,114,115]. Several studies reported that HN can activate various signaling pathways such as JAK2-STAT3, p38mitogen-activatedprotein kinases MAPK P38MAPK), AMP-activated protein kinase (AMPK) and ERK1/2 and also c-JunN-terminalkinase (JNK) which are involved in cell proliferation and protect neuronal cells from apoptosis [116,117,118,119,120,121]. A recent study suggest that primary human fibroblasts exposed to HN modestly increased mitochondrial respiration and selected components of the senescence-associated secretory phenotypes (SASPs) in doxorubicin-induced senescent cells partially via the JAK pathway [122]. HN blocks pro-apoptotic Bax by prevention of its translocation from the cytosol into mitochondria and suppression of cytochrome C release [122]. Similarly, our findings also suggest that HN suppresses AgNPs-induced activation of Bax. Further, our studies demonstrated that HN blocks AgNPs-induced loss of viability and cell proliferation by mitigation of level of LDH, ROS, LPO, NO and also it increases the level of anti-oxidants and eventually leads to prevention of cell death. Based on our findings and previous studies, we proposed a model to describe activation /deactivation of various mechanistic pathways by HN in human neuroblastoma cells (Figure 13).

## 3. Materials and Methods

### 3.1. Cell Lines and Reagents

HN was purchased from Peptide Institute Inc., Japan. Silver nitrate was procured from Sigma-Aldrich (St. Louis, MO, USA). Penicillin-streptomycin, trypsin-EDTA, DMEM cell culture medium, fetal bovine serum (FBS), and antibiotic-anti-mycotic reagents were obtained from Life Technologies/Gibco (Grand Island, NY, USA). The in vitro toxicology assay kit was purchased from Sigma-Aldrich. The reagent kits for the measurement of malondialdehyde (MDA), protein carbonyl content, and antioxidant assay kits were purchased from Sigma-Aldrich. All other chemicals were purchased from Sigma-Aldrich unless otherwise stated.

### 3.2. Synthesis and Characterization of AgNPs

AgNPs synthesis was performed according to a previously described method [34]. Briefly, 1.0 mg/mL delphinidin was dissolved in methanol, AgNO_3_ was dissolved in water at a concentration 5 mM, and both delphinidin and AgNO_3_ were incubated at 40 °C for 6 h. Further characterization of the synthesized AgNPs was performed as previously described [123]. The bioreduction of silver ions was monitored spectrophotometrically between 300 and 600 nm. The reduced silver solution was sonicated for 10 min to separate silver nanomaterials from the biomolecules. After sonication, the solution was filtrated with a 0.2-µm syringe filter. The reduced silver metal was purified by repeated centrifugation at 15,000 rpm for 30 min and the pellets were washed with distilled water to remove the impurities. Purified AgNPs were characterized using various analytical techniques such as UV-vis spectroscopy, X-ray diffraction (XRD), Fourier transform infrared spectroscopy (FTIR), dynamic light scattering (DLS), and transmission electron microcopy (Tokyo, Japan).

### 3.3. Cell Culture and Treatments

The human neuroblastoma cell line SH-SY5Y was obtained from ATCC. SH-SY5Y cells were cultured in DMEM containing 10% FBS, 100 U/mL penicillin, and 100 mg/mL streptomycin and were incubated in a humidified atmosphere with 5% CO_2_ at 37 °C. All cells were cultured in 75-cm2 tissue culture flasks (Corning, NY, USA) at 37 °C in the presence of 5% CO_2_ and 95% relative humidity. The cells were sub-cultured, usually twice a week, with 1 × 106 viable cells/mL and incubated at 37 °C in a 5% CO_2_ atmosphere. The medium was replaced the next day with fresh media, and the cells were further incubated for 24 h prior to HN or AgNPs exposure. Experiments were performed in 96-, 24-, and 12-well plates and 100-mm cell culture dishes, as required. Cells were treated with various concentrations of HN or AgNPs for 24 h. To study the protective effect of HN, cells were pre-treated with HN (10 μg/mL) for 24 h, followed by AgNPs (10 μg/mL) treatment in the continued presence of HN (10 μg/mL) for an additional 24 h. Cells cultured in medium without the addition of HN or AgNPs were used as the control.

### 3.4. Cell Viability Assay

Cell viability was measured using cell counting kit-8 (CCK-8; CK04-01, Dojindo Laboratories, Kumamoto, Japan). Briefly, HN or AgNPs cells were plated in 96-well flat-bottom culture plates containing various concentrations of HN and/or AgNPs. After 24-h culture at 37 °C in a humidified 5% CO_2_ incubator, the CCK-8 solution (10 μL) was added to each well, and the plate was incubated for another 2 h at 37 °C. The absorbance was measured at 450 nm using a microplate reader (Multiskan FC; Thermo Fisher Scientific Inc., Waltham, MA, USA).

### 3.5. BrdU Cell Proliferation Assay

Cell proliferation was determined according to manufacturer’s instructions (Roche). SH-SY5Y cells were pretreated with or without 10 μg/mL HN for 24 h. Cells were then treated with 10 μg/mL HN and/or 10 μg/mL AgNPs for 24 h and BrdU labeling solution was added to the culture medium and incubated for 2 h. The cells were fixed and the level of incorporated BrdU was determined using the BrdU enzyme-linked immunosorbent assay (ELISA) kit (Roche, Santa Clara, CA, USA) following the manufacturer’s instructions. Proliferation of the untreated cells at 0 h was considered 100%.

### 3.6. Cell Death Measurement

SH-SY5Y cells were plated in six-well plates (2 × 105 cells per well) and incubated with or without 10 μg/mL HN for 24 h. Cells were then treated with 10 μg/mL HN and/or 10 μg/mL AgNPs for 24 h. Cells were also cultured in medium without the addition of either HN or AgNPs (10 μg/mL) and used as the control.

### 3.7. Assessment of Membrane Integrity

The membrane integrity of SH-SY5Y cells was evaluated using a lactate dehydrogenase (LDH) cytotoxicity detection kit (Sigma-Aldrich-St, Louis, MO, USA). Briefly, SH-SY5Y cells were pretreated with or without 10 μg/mL HN for 24 h. Cells were then treated with 10 μg/mL HN and/or 10 μg/mL AgNPs for 24 h. Subsequently, 100 μL of cell-free supernatant from each well was transferred in triplicate into the wells of a 96-well plate, and 100 μL of the LDH reaction mixture was added to each well. After 3 h of incubation under standard conditions, the optical density of the final solution was determined at a wavelength of 490 nm using a microplate reader.

### 3.8. Assessment of Dead-Cell Protease Activity

A dead-cell protease activity assay was performed according to the method described earlier [34]. SH-SY5Y cells were pretreated with or without 10 μg/mL HN for 24 h. Cells were then treated with 10 μg/mL HN and/or 10 μg/mL AgNPs for 24 h. The protease activity was determined by assessing the association of intracellular proteases with a luminogenic peptide substrate (alanyl-alanylphenylalanyl-aminoluciferin). Luminogenic peptide substrate (5 μL) was added to each well, and luminescence was measured to determine the number of dead cells. The peptide substrate was incubated for 15 min at 37 °C. The luminescence was measured with a luminescence counter (Perkin Elmer, Waltham, MA, USA). The degree of luminescence reaction measured in this assay reflected the dead-cell protease activity.

### 3.9. Determination of ROS, MDA, Nitric Oxide (NO), and Carbonylated Protein Levels

ROS was estimated as described previously [123]. SH-SY5Y cells were pretreated with or without 10 μg/mL HN for 24 h. Cells were then treated with 10 μg/mL HN and/or 10 μg/mL AgNPs for 24 h. After washing twice with phosphate-buffered saline (PBS), the cells were supplemented with 20 μM DCFH2-DA, and incubation was continued further for 30 min at 37 °C. The cells were rinsed with PBS, 2 mL PBS was added to each well, and the fluorescence intensity was determined using a Gemini EM spectrofluorometer (Molecular Devices, Sunnyvale, CA, USA) at an excitation wavelength of 485 nm and an emission wavelength of 530 nm.

MDA levels were determined using a thiobarbituric acid-reactive substances assay as previously described with suitable modifications [123]. NO production was spectrophotometrically quantified using Griess reagent (Sigma-Aldrich). The absorbance was measured at 540 nm, and nitrite concentration was determined using a calibration curve prepared with sodium nitrite as the standard [123]. Carbonylated protein content was measured according to a previously described method [123].

### 3.10. Measurement of Anti-Oxidative Marker Levels

The expression levels of oxidative and anti-oxidative stress markers were determined as described previously [123]. SH-SY5Y cells were pretreated with or without 10 μg/mL HN for 24 h. Cells were then treated with 10 μg/mL HN and/or 10 μg/mL AgNPs for 24 h. The levels of anti-oxidative stress markers, reduced glutathione (GSH), thioredoxin (TRX), catalase (CAT), superoxide dismutase (SOD), glutathione peroxidase (GPx) and glutathione S-transferase (GST) were determined according to the manufacturer’s instructions. The cells were harvested in chilled PBS by scraping and washing twice with 1× PBS at 4 °C for 6 min each at 1500 rpm. The cell pellet was sonicated at 15 W for 10 s (three cycles) in order to obtain the cell lysate. The resultant supernatant was stored at −70 °C until further analysis.

### 3.11. Determination of Mitochondrial Membrane Potential (MMP)

MMP was measured according to the manufacturer’s instructions (Molecular Probes, Eugene, OR, USA) using the cationic fluorescent indicator, JC-1 (Molecular Probes). SH-SY5Y cells were pretreated with or without 10 μg/mL HN for 24 h. Cells were then treated with 10 μg/mL HN and/or 10 μg/mL AgNPs for 24 h and then cells were incubated with 10 μM JC-1 at 37 °C for 15 min, washed with PBS, resuspended in PBS, and then the fluorescence intensity was measured. MMP is expressed as the ratio of the fluorescence intensity of the JC1 aggregates to that of the monomers.

### 3.12. Measurement of ATP Level

The ATP level was measured according to the manufacturer’s instructions (Catalog Number MAK135; Sigma-Aldrich). SH-SY5Y cells were pretreated with or without 10 μg/mL HN for 24 h. Cells were then treated with 10 μg/mL HN and/or 10 μg/mL AgNPs for 24 h. Decreased levels of ATP indicated increased cytotoxicity to the treated cells.

### 3.13. Analysis of Mitochondrial DNA Copy Number

A mitochondrial dysfunction analysis was carried out by assessing the mitochondria copy number using real-time PCR amplification. SH-SY5Y cells were pretreated with or without 10 μg/mL HN for 24 h. Cells were then treated with 10 μg/mL HN and/or 10 μg/mL AgNPs for 24 h. To determine the copy number, the following primers were used: mtDNA forward primer, CCTATCACCCTTGCCATCAT; mtDNA reverse primer, AGGCTGTTGCTTGTGTGAC. To quantify the nuclear DNA, we used a primer set that detects the Pecam gene on chromosome 6: nuclear DNA, the following primers were used: forward primer, ATGGAAAGCCTGCCATCATG; reverse primer, TCCTTGTTGTTCAGCATCAC [90].

### 3.14. TUNEL Analysis

For the detection of apoptotic cells, SH-SY5Y cells were pretreated with or without 10 μg/mL HN for 24 h. Cells were then treated with 10 μg/mL HN and/or 10 μg/mL AgNPs for 24 h, following which the terminal deoxynucleotidyl transferase-mediated dUTP nick end labelling (TUNEL) method was employed, using an in situ detection kit (Promega, Madison, WI, USA) and by following the manufacturer’s instructions.

### 3.15. Measurement of 8-Oxo-7,8-Dihydro-2′-Deoxyguanosine (8-Oxo-dG) and 8-Oxo-G Levels

SH-SY5Y cells were pretreated with or without 10 μg/mL HN for 24 h. Cells were then treated with 10 μg/mL HN and/or 10 μg/mL AgNPs for 24 h. 8-oxo-dG and 8-oxo-G contents was determined as described previously [34,123,124] and by following the manufacturer’s instructions (Trevigen, Gaithersburg, MD, USA).

### 3.16. Reverse Transcription-Quantitative Polymerase Chain Reaction (RT-qPCR)

RT-qPCR was performed according to a method described earlier [27]. SH-SY5Y cells were pretreated with or without 10 μg/mL HN for 24 h. Cells were then treated with 10 μg/mL HN and/or 10 μg/mL AgNPs for 24 h. Total RNA was extracted using the PicoPure RNA isolation kit (Arcturus Bioscience, Mountain View, CA, USA). Samples were prepared according to the manufacturer’s instructions. RT-qPCR was conducted using a Vill7 device (Applied Biosystems, Foster City, CA, USA) and SYBR Green was used as the double-stranded DNA-specific fluorescent dye (Applied Biosystems). Target gene expression levels were normalized to glyceraldehyde-3-phosphate dehydrogenase (*GAPDH)* expression, which was unaffected by the treatment. The sequences of the PCR primers are as shown in Table 1. 

### 3.17. Statistical Analysis

Independent experiments were repeated at least thrice, and data are represented as mean ± standard deviation (SD) for all triplicates within an individual experiment. Data were analyzed using a *t*-test, multivariate analysis, or one-way analysis of variance (ANOVA), followed by Tukey’s test for multiple comparisons to determine the differences between groups (denoted by an asterisk). GraphPad Prism 8 analysis software was used for all analysis.

## 4. Conclusion

AgNPs are the most extensively used nanomaterials among other metallic nanoparticles as antimicrobial and anticancer agents. This ubiquity leads to inevitable exposure of human beings to these particles in everyday life. However, the effects of AgNPs on neuronal cells are still largely unknown. In this study, we used human neuroblastoma cancer cells as a cellular model to study the neurotoxicity. HN is a mitochondrial peptide that exhibits cytoprotective and neuroprotective effects against a variety of cell types. Therefore, the present study was undertaken to evaluate the effect of HN against AgNPs-induced neurotoxicity in SH-SY5Y cells. AgNPs were synthesized using delphinidin as the reducing and stabilizing agent. The prepared AgNPs were characterized using various analytical techniques. The SH-SY5Y cells exposed to AgNPs underwent dose-dependent cell death, increased reactive oxygen species generation, increased level of leakage of lactate dehydrogenase, dead cell protease activity, and loss of plasma-membrane integrity, thus indicating the cytotoxic potential of AgNPs. Oxidative and nitro-oxidative stress induced by AgNPs was found to lead to mitochondrial dysfunction, ER stress, DNA damage, and apoptosis. These data supported the fact that AgNPs can potentially induce neurotoxicity in SH-SY5Y cells. Pretreatment with HN or co-treatment with HN and AgNPs competently inhibited AgNPs-induced ROS formation, cell death, loss of membrane integrity, and mitochondrial dysfunction. HN pretreatment decreased the level of oxidative markers such as ROS, MDA, NO, and carbonylated proteins and increases the level of antioxidants. Furthermore, HN protected mitochondrial dysfunction by rescuing mitochondrial membrane potential, increases mitochondrial DNA copy number, increases the expression of the central regulator of biogenesis of mitochondria, called PGC1 alpha, and upregulates the expression of Bcl-2. Finally, HN pretreatment with AgNPs prevented oxidative stress-induced DNA damage. So far, no study has demonstrated the protective effect of HN on AgNPs induced neurotoxicity. Our study demonstrated that AgNPs-induced neurotoxic effects can be reversed by the co-administration of HN with AgNPs, suggesting that oxidative stress is the major cause of AgNPs-mediated neurotoxicity; thus, HN could potentially be used as a neuroprotective agent against AgNPs induced neurotoxicity. Furthermore, our data are the first to show that HN has a protective effect on AgNPs-induced apoptosis of SH-SY5Y cells; however, more specific mechanisms need to be clarified. Considering all these beneficial effects of HN against neurotoxicity caused by AgNPs, new avenues to develop effective strategies to reduce the neurotoxicity and to develop biocompatible nanomaterials could be opened up.

## Figures and Tables

**Table 1:** The sequences of the PCR primers.

Gene	List of Primers
PGC1 alpha	F: CAATGAATGCAGCGGTCTTA
	R: ACGTCTTTGTGGCTTTTGCT
IRE1	F: GACAGGCTCAATCAAATGG
	R: CGGTCAGGAGGTCAATAACA
PERK	F: ATTGCATCTGCCTGGTTAC
	R: GACTCCTTCCTTTGCCTGT
ATF6	F: CAGGGAGAAGGAACTTGTGA
	R: ACTGACCGAGGAGACGAGA
Caspase-3	F: AGGGGTCATTTATGGGACA
	R: TACACGGGATCTGTTTCTTTG
Bax	F: CGAGCTGATCAGAACCATCA
	R: GAAAAATGCCTTTCCCCTTC
Bcl-2	F: TAAGCTGTCACAGAGGGGCT
	R: TGAAGAGTTCCTCCACCACC
GAPDH	F: AGGTCGGTGTGAACGGATTTG
	R: TGTAGACCATGTAGTTGAGGTCA
